# Codon Usage and Adenovirus Fitness: Implications for Vaccine Development

**DOI:** 10.3389/fmicb.2021.633946

**Published:** 2021-02-10

**Authors:** Judit Giménez-Roig, Estela Núñez-Manchón, Ramon Alemany, Eneko Villanueva, Cristina Fillat

**Affiliations:** ^1^Institut d’Investigacions Biomèdiques August Pi i Sunyer (IDIBAPS), Barcelona, Spain; ^2^Procure Program, Institut Català d’Oncologia- Oncobell Program, IDIBELL, L’Hospitalet de Llobregat, Barcelona, Spain; ^3^Cambridge Centre for Proteomics, Department of Biochemistry, University of Cambridge, Cambridge, United Kingdom; ^4^Centro de Investigación Biomédica en Red de Enfermedades Raras (CIBERER), Barcelona, Spain; ^5^Facultat de Medicina i Ciències de la Salut, Universitat de Barcelona (UB), Barcelona, Spain

**Keywords:** codon optimization, viral attenuation, live-attenuated vaccines, adenovirus-based vaccines, codon usage bias

## Abstract

Vaccination is the most effective method to date to prevent viral diseases. It intends to mimic a naturally occurring infection while avoiding the disease, exposing our bodies to viral antigens to trigger an immune response that will protect us from future infections. Among different strategies for vaccine development, recombinant vaccines are one of the most efficient ones. Recombinant vaccines use safe viral vectors as vehicles and incorporate a transgenic antigen of the pathogen against which we intend to generate an immune response. These vaccines can be based on replication-deficient viruses or replication-competent viruses. While the most effective strategy involves replication-competent viruses, they must be attenuated to prevent any health hazard while guaranteeing a strong humoral and cellular immune response. Several attenuation strategies for adenoviral-based vaccine development have been contemplated over time. In this paper, we will review them and discuss novel approaches based on the principle that protein synthesis from individual genes can be modulated by codon usage bias manipulation. We will summarize vaccine approaches that consider recoding of viral proteins to produce adenoviral attenuation and recoding of the transgene antigens for both viral attenuation and efficient viral epitope expression.

## Introduction

Adenoviruses are non-enveloped viruses with a double-stranded DNA genome. They were first discovered in 1953 by Rowe and colleagues (Huebner et al., 1954). Adenoviruses are broadly present across vertebrate species and have been classified into five genera: Mastadenovirus, Aviadenovirus, Siadenovirus, Atadenovirus, and Ichtadenovirus. Mastadenoviruses infect mammals and are classified into 7 species (A–G) (Seto et al., 2011; Dhingra et al., 2019). None causes severe diseases in humans and, therefore, they are not considered a serious health hazard.

**FIGURE 1:**
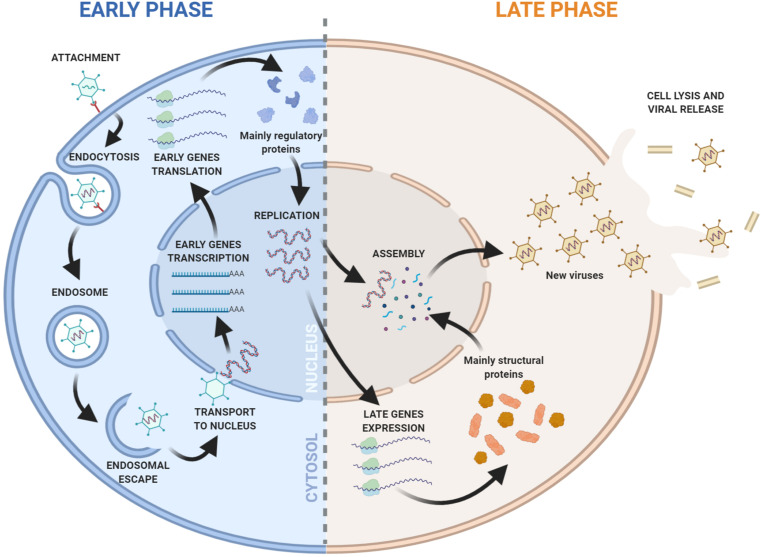
Overview of the Adenovirus replication cycle. Early and late phases of the cycle are indicated. The viral DNA replication marks the progression from early to late transition.

**FIGURE 2:**
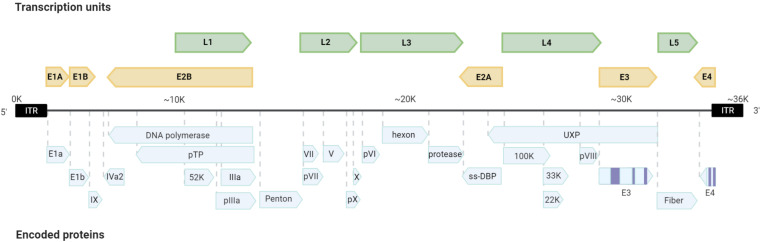
Scheme of the adenoviral genome. The organization of the transcription units and encoded proteins are presented.

Adenoviruses have icosahedral capsids of 70–100 nm. They are composed of 252 capsomeres, with 12 pentons. Each penton contains a fiber. The capsid fiber is responsible for the recognition and interaction with the host cell. Adenoviral replication and transcription take place in the nucleus of the host infected cell where viral DNA is first translocated through the nuclear pore associated with its core proteins (Charman et al., 2019; Figure 1). Adenoviruses have mid-sized DNA genomes (30–40 kb), with genes encoded in both strands, and an extensive use of alternative splicing (Donovan-Banfield et al., 2020). Adenoviruses present highly orchestrated sequential gene expression, with early (E1A, E1B, E2A, E2B, E3, and E4) and intermediate (IX and IVa) transcription units mostly displaying regulatory functions. Early genes are also responsible for inducing the Major Late Promoter, which activates the transcription of late expression units (L1, L2, L3, L4, and L5) that are processed by alternative splicing and encode the structural proteins of the new virions (Figure 2).

Human adenoviruses exhibit a highly variable tissue tropism, including the respiratory tract, gastrointestinal tract and conjunctiva. Infections are typically caused by inhalation of aerosolized droplets from infected individuals, conjunctival inoculation, or fecal-oral contact. They usually cause mild symptomatology such as cold-like symptoms, fever, sore throat, acute bronchitis, pneumonia, diarrhea, or conjunctivitis in patients. Even they are considered non-hazardous for the general population, there is an FDA approved vaccine against adenoviruses, consisting on an oral administration of live Ad4 and Ad7 serotypes, exclusively used for the US military personnel. This vaccine was developed to control an endemic adenovirus infection at military training camps resulting in the near elimination of Ad4/7 related disease (Broderick et al., 2017). Currently, there is no approved vaccine for the general population (Kuschner et al., 2013; Lion, 2014) with an ongoing debate on whether it would be of general interest to develop a vaccine for public use.

However, there is a growing interest for using adenoviruses as scaffolds in vaccine production to fight a broad repertoire of pathogens. Several key properties make them good candidates: infect dividing and non-dividing cells, can be delivered both systemically, or through mucosal surfaces, induce both innate and adaptive immunity, follow a lytic cycle and are physically and genetically stable. Even more importantly, adenoviruses are easy to genetically engineer, allow high levels of transgene expression, and are able to grow to high titers *in vitro* (Gao et al., 2019). Adenovirus infection triggers the release of cytokines, generating an inflammatory milieu in the infected tissue. This is the consequence of the virus recognition by Pathogen Recognition Receptors or the specific interaction between the adenoviral fiber and the CAR receptor on the cell surface, activating a cascade of events that induce cytokine release (Majhen et al., 2014). In the infected cell, adenoviral replication and viral protein expression when using replication-competent vectors triggers B cell and CD4 + and CD8 + T cell responses against viral and transgene proteins. This immune response can be a beneficial effect in the vaccine context, where adenoviruses can act as a natural adjuvant (Robert-Guroff, 2007). On the other hand, a balanced innate immune response seems optimal to elicit immunity as the induction of very high levels of type I interferon upon dendritic cell infection can downregulate transgene expression and the formation of optimal antibody responses (Hensley et al., 2007).

## Adenovirus-Based Vaccines

**FIGURE 3:**
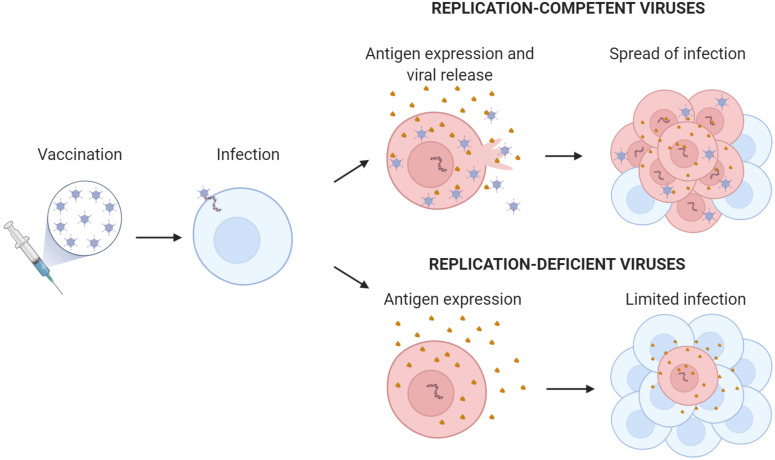
Replication-competent versus replication-deficient adenovirus encoding transgenic antigen. The scheme illustrates the amplification effect of replication-competent adenovirus with the generation of new virions and increased antigen production. Antigen expressed from the adenovirus is shown in brown.

Adenovirus-based vaccines use replicating or non-replicating virus as platforms to express the vaccinating antigen or epitope (Figure 3). The antigen in the formulated adenovirus vaccines can be present as a protein or as a transgene. If the adenovirus contains the antigen as a protein, there is no need for gene expression and the virus can be immunogenic even in the absence of infectivity or transduction. Usually the virus is engineered to display the antigen fused to a viral structural protein of the capsid such as the hexon or the fiber (Vujadinovic and Vellinga, 2018). This antigen display on the capsid allows for the direct presentation of the antigen to B-cell receptors (membrane antibodies) and the indirect presentation to T-cell receptors. For the presentation and activation of T cells, the virus is internalized and processed by antigen-presenting cells (APCs) that load viral and vaccinating antigen peptides in the MHC-I and MHC-II. Antigen display circumvents one of the main limitations of viral vaccines: the presence of pre-existing neutralizing antibodies that opsonize the virus and prevent infection and gene expression. However, this approach is limited by the amount of exogenous protein that the hexon or the fiber can accommodate, restricting it to approximately 100 amino acids. Thus, most of the conformational B-cell epitopes that are not formed by contiguous protein sequences cannot be displayed appropriately in the adenovirus capsid to generate antibodies. As an alternative, the antigen can be encoded as a gene in the virus. This approach requires the transgene expression in APCs or in other cells that can indirectly provide the expressed antigen to APCs. Once infected with adenoviruses, humans develop neutralizing antibodies that preclude viral infection and, consequently, transgene expression. In these seropositive hosts, the gene-expression vaccine will not be effective. To solve this limitation, vaccines can be developed from rare or low-prevalent human serotypes such as HAd2, HAd26, HAd35, HAd48, or HAd64 or from adenoviruses from non-human primates (Bots and Hoeben, 2020; Garofalo et al., 2020). Nevertheless, in the context of replication-competent vaccines, employing the first strategy seems the safest option for vaccination, as non-human virus zoonoses could potentially arise from the use of adenoviruses from other animals (Bots and Hoeben, 2020; Garofalo et al., 2020). Therefore, improving viruses such as HAd5 is a promising strategy in adenoviral-based vaccine development. Viral shielding of Ad5 capsids with a variety of nanomaterials such as PEG, or PBAEs among others have shown to provide protection toward pre-existing Ad neutralizing antibodies (Hong and Yun, 2019; Brugada-Vilà et al., 2020).

Another key issue to address when considering adenovirus-based vaccines, as well as other virus-vectored vaccines, is the viral immunodominance. A strong response against the virus epitopes can mask the response against the vaccinating antigen (Schöne et al., 2017). This occurs in naïve and in previously exposed hosts. When the host has been previously exposed to the virus and there are pre-existing memory B and T cells immunodominance is aggravated. This immunodominance was noted in a vaccination trial against HIV using adenoviruses (Frahm et al., 2012). Moreover, eliciting a memory response will further dominate the breath of epitopes recognized. Again, this can be solved using rare low-prevalent serotypes or non-human adenoviruses, but cross-reactive immunity has been observed that may reduce the efficacy of this approach (Frahm et al., 2012). When repeated vaccination is required, a classical strategy to palliate the viral vector immunodominant memory response is the use of a different virus expressing the same antigen in the second dose of the vaccine (the boost), known as heterologous prime-boost. However, even in naïve hosts the virus can still be immunodominant compared to the vaccinating antigen (Schirmbeck et al., 2008). Immunodominance is associated to a competition of responding immune cells for adenoviral epitopes and antigen recognition. Several factors contribute to immunodominance (Sadegh-Nasseri and Kim, 2019; Abbott and Crotty, 2020). One key factor is the amount of antigen produced (Quinn et al., 2015). Avoiding adenovirus gene expression using gutless vectors as vaccines can prevent virus immunodominance (Kron et al., 2011). In humans, immunodominant epitopes for B and T cells are mainly present in capsid proteins, namely the penton, hexon and fiber (Tischer et al., 2016). Codon usage can be used as a strategy to increase the efficacy of a replication-competent vaccine by increasing the relative levels of the vaccinating antigen compared to the levels of these immunodominant viral proteins but without decreasing virus gene expression to preserve virus replication.

Successful recombinant vaccines based on the use of replication-deficient adenoviruses have been developed over the last years (reviewed in Majhen et al., 2014; Barouch et al., 2015). Early developments were based on the deletion of the E1 region. Further improvements in vectorization resulted with the deletion of E1 and E3 regions what allows to accommodate transgenes of approximately 7.5 Kb. Such vectors are stable, easy to manipulate can be produced at high titers and they have shown safety and immunogenicity in clinical trials (Coughlan, 2020).

Nowadays, during the SARS-CoV-2 pandemic, several replication-defective adenoviruses expressing the spike glycoprotein of the SARS-CoV-2 are in development. One of them, based on the HAd5, (clinicaltrials.org, NCT04341389) has shown tolerability and immunogenicity at 28 days post-vaccination and could induce humoral and T cell immune responses, activating both CD4 + and CD8 + T cells (Zhu et al., 2020). Interestingly, the Ad5-nCoV vaccine has shown a protective effect against SARS-CoV-2 challenge in wild-type BALB/c mice and ferrets following intranasal immunizations, highlighting the potential of the mucosal vaccination route (Wu et al., 2020). A chimpanzee adenovirus-vectored vaccine (ChAdOx1 nCoV-19) administered to 1,077 participants showed an acceptable safety profile with the induction of both humoral and cellular immune responses (Folegatti et al., 2020). Another adenovirus-based vaccine against SARS-CoV-2 is under development using Adenovirus serotype 26 (Ad26.COV2.S) and it has shown that a single shot of the vaccine induces protection in non-human primates (Mercado et al., 2020). At present, this vaccine is being evaluated in clinical trials. A fourth adenovirus vaccine against SARS-CoV-2 is ongoing, which consists of two non-replicative adenoviruses based on Ad26 and Ad5 expressing the spike glycoprotein (S) and implemented in a prime-boost regimen. The vaccine was well tolerated and produced humoral and cellular immune responses in healthy adults (Logunov et al., 2020).

Despite the potential interest on replication-deficient adenovirus-based vaccines, obtaining immuno-protection usually requires high viral doses to elicit immunity. Adenovirus vaccines based on fully-replicative viruses are able to expand antigen expression and trigger improved immunity. However, the potential of replication-competent adenoviruses as vectors for immunization has been poorly explored to date. To preserve safety, replicative-competent adenovirus vaccines need to be attenuated to avoid causing pathologies associated with adenoviral infections. Therefore, a good balance between antigen-transgene expression and adenoviral replication is crucial to develop a successful vaccine.

Live attenuated viruses are typically variants of the wild-type virus that have a reduced capacity to grow. Historically, attenuation was achieved by culturing viruses using cells from other species. Over time the virus would gain mutations allowing it to better replicate in the new host. If administered again to humans, the newly acquired mutations would then have a detrimental effect on viral replication resulting in a human attenuated virus (Smith, 2012). This technique was time-consuming and made it difficult to select and isolate the proper attenuated candidate.

More rational strategies for virus attenuation in vaccine development have been in the spotlight of many researchers in recent years as they allowed for more efficient, specific, and safe vaccine development. Strategies based on genetic engineering of the viral genome have been studied. A group of these strategies include the deletion of dispensable transcription units. This also facilitates the accommodation of transgenic antigens, since for efficient viral packaging, the insertion of foreign sequences into the intact adenoviral genome is limited to around 1.8 Kb. Deletion of E3, which encodes gene products non-essential for viral replication has been commonly used. In these vaccines, the E3 promoter or the Major Late Promoter if splice acceptors are maintained or even stronger promoters such as the CMV drive the expression of the transgenes. One example is the Ad4-H5-Vtn vaccine in which the expression of the influenza hemagglutinin gene in the E3-deleted adenovirus serotype 4 genome induced protective immunity against H5N1 influenza virus infection in mice intranasally immunized (Alexander et al., 2012). Oral vaccination with Ad4-H5-Vtn, in a phase I clinical trial, resulted in no serious adverse event suggesting its potential as a priming vaccine (Gurwith et al., 2013). Another example of attenuation of replicative- competent adenoviruses by means of viral gene deletion is the replication-competent Ad26 with the E3 and E4 regions deleted generated as vector to develop candidate HIV-1 vaccines (Maxfield et al., 2015). Which deletion is most suitable for an adenovirus vaccine may depend on different variables such as the size of the insert or the virus serotype among others.

Additional strategies for viral attenuation rely on genome modifications to restrict adenoviral replication to specific cells by the use of miRNAs or tissue-specific promoters to modulate viral gene expression and tropism or the retargeting of adenovirus toward dendritic cells to increase antigen presentation (Tatsis and Ertl, 2004; Bofill-De Ros et al., 2015). In general, replication-competent adenovirus vaccines lead to high transgene expression and require lower doses to confer persistent immunity. However, a good balance between the amount of the vaccinating antigen and the immunodominance of viral epitopes needs to be achieved to generate specificity in the immune response. In this regard, single-cycle replicating adenovirus have shown to amplify transgene expression. These vaccines replicate its DNA, and the transgene but incorporate a deletion that does not permit virion production (Crosby et al., 2015). Alternatively, codon optimization of viral proteins and transgenes in order to maximize transgene expression while controlling adenoviral protein expression could be considered as a novel approach. This could boost adenovirus-based vaccine development opportunities.

## Synonymous Gene Recoding for Viral Attenuation in Adenovirus Vaccine Development

The genetic code is redundant, with eighteen out of twenty amino acids being encoded by two, four, or six synonymous codons. The frequency in which synonymous codons are used differs from what would be expected by chance, with some being rarely used and others more frequently. This phenomenon, known as codon usage bias, happens to be species-specific (Grantham et al., 1980; Pouwels and Leunissen, 1994) and is described to correlate with the relative abundance of iso-accepting tRNAs (i.e., the tRNAs that are associated with the same amino acid but recognize different codons). The correlation between specific tRNA abundance and codon usage could determine how efficiently particular transcripts are translated. In this way, codon optimality is correlated with how efficiently codons are decoded by ribosomes and thus, protein synthesis levels. The third base position in a codon (GC3) or (AT3) has been recently linked to human mRNA stability showing that GC3 codons stabilize mRNA while AT3 codons destabilize mRNA. Thus leading to consider GC3 codons and AT3 codons as optimized and de-optimized, respectively (Hia et al., 2019). Due to the inherent viral dependence on cellular translational machinery, viral codon usage is, in many cases, considered as the result of mutational and selective pressure determined by the tRNA pool of the host cell (Duret, 2002; Das et al., 2005; Chamary et al., 2006; Hershberg and Petrov, 2008).

Substitution of synonymous codons based on the differential codon usage in a given organism has been used as a strategy to optimize or deoptimize the expression of viral proteins. Altering the codon usage of a virus by codon deoptimization has been widely explored for vaccine development against HIV, Lassa Virus, or Influenza virus among others (Mueller et al., 2006; Coleman et al., 2008; Cai et al., 2020). Codon deoptimization of certain genes in the viral genome requires the use of less abundant or rare tRNAs within the host tRNA pool, leading to slower translation rates (Zhou et al., 1999; Jack et al., 2017). This, results in a lower replicative viral fitness while maintaining a strong immune response and, due to the numerous mutations used to recode the engineered genes, avoiding reversion to virulence. Furthermore, this approach can be extended to consider not only the bias in codon usage, but codon pair bias, which considers that there are higher chances to find certain codons next to specific codons (Gutman and Hatfield, 1989; Coleman et al., 2008; Gamble et al., 2016). In this line, polioviruses for example have been attenuated by recoding their genomes with underrepresented codon pairs (Coleman et al., 2008). This attenuation strategies can work for both DNA and RNA viruses and are commonly used nowadays (Martínez et al., 2019).

Despite of the fact that codon deoptimization is a well-established general strategy for viral attenuation, there are no adenovirus-based vaccines attenuated via codon recoding. In fact, recent studies suggest that codon optimization, instead of deoptimization, could be a potential strategy for adenovirus attenuation (Villanueva et al., 2016). In the context of replication-competent vectors, the codon recoding strategy should take into account an attenuated virus replication and the relative increase in antigen production compared to viral proteins. Experimental results after codon optimization of the adenoviral fiber in a non-viral system showed increased protein expression, reinforcing the importance of using optimal codons adapted to the host codon usage for efficient gene expression in non-viral contexts. Conversely, codon-optimized fiber expression, as well as the expression of other late proteins, were attenuated in a viral context, impairing viral fitness, thus leading to an attenuated adenovirus. We speculate that this attenuation is the result of a competition for a limiting pool of aminoacylated-tRNAs. Aminoacylated-tRNAs could then act as a limiting resource during the late phase of the viral replication cycle, which involves exceptionally high gene expression levels (Villanueva et al., 2016).

**FIGURE 4:**
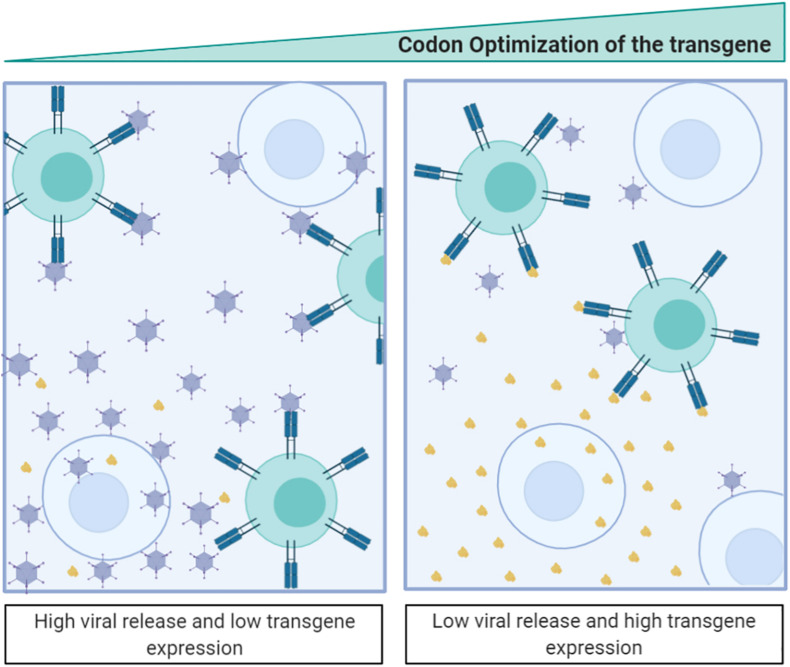
Antigen-transgene recoding strategy for adenoviral-based vaccines. The scheme illustrates that codon optimization of the transgenic antigen induces its high expression while attenuates adenovirus fitness. Antigen expressed from the adenovirus is shown in brown.

Following this argument, the introduction of transgenes using the same codons as other structural proteins could generate an intergenic competition resulting in the loss of viral fitness (Villanueva et al., 2016). Indeed, an extreme optimization of a transgene could break the balance between the overall codon usage and the tRNA concentrations and affect the expression of other genes (Qian et al., 2012). This is considered detrimental for virotherapy and links to the observation that in most clinical trials involving armed oncolytic viruses (where the aim is to obtain high levels of transgene and viral particles) the codon usage of the transgene is suboptimal (Núñez-Manchón et al., 2020). We have recently targeted this question showing that, when expressing highly optimized transgenes, the transgene is highly expressed during the first round of infection, but its expression negatively impacts viral fitness (Núñez-Manchón et al., 2020). We have further shown how, by deoptimizing the transgene codon usage, both the viral and transgene protein expression can be balanced, generating highly replicative antitumor viruses expressing therapeutic genes. From the adenoviral-based vaccine-development perspective, intergenic competition opens new and exciting opportunities where the transgene expression can be boosted while attenuating adenoviral replication. Therefore, optimization of the codon usage of the transgene could have a double impact resulting both in viral attenuation and in an increase of the engineered epitope expression (Figure 4).

Protein recoding to modify either viral proteins or transgene antigens should carefully consider that synonymous codon changes may affect protein conformation and stability or even alter protein function. Synonymous codons have been shown to contribute differently to the secondary structures of the resulting protein (Buhr et al., 2016) and most complex structures may contain non-preferred codons that are translated slower and, therefore, allow ordered and sequential folding of the resulting protein. Therefore, when recoding viral genes, or other viral epitopes for vaccine development, the immunogenicity of the new engineered gene needs to be carefully determined.

Since tRNA availability is not the unique factor influencing protein expression, several other aspects concerning gene recoding are key for vaccine development (Brule and Grayhack, 2017). For example, the increase of CpG dinucleotides frequency in both coding and non-coding regions has also been demonstrated to attenuate RNA viruses early after cell entry by preventing their replication (Burns et al., 2009; Atkinson et al., 2014; Simmonds et al., 2015). Although little is known about the biological mechanism that leads to attenuation, this last strategy seems to be less effective than codon deoptimization (Groenke et al., 2020), probably since it prevents a high expression of the viral genes and any engineered transgene epitope.

Finally, attenuation of adenoviruses can have further implications helping to overcome anti-adenovirus vector immunity. Even if the immunogenicity of the adenovirus is desirable since it can act as adjuvant triggering inflammation, a too strong immunity could interfere with the specific antigen immune response of the vaccine reducing their efficacy. Thus, attenuation of adenovirus based-vaccines with synonymous genes recoding of viral proteins or transgene-epitopes is a promising strategy to favor specific antigen-transgene immune response.

Codon optimization of the transgenic antigen in replication-deficient adenovirus is a tested approach for increasing protein expression. A recent example of this strategy is the prophylactic vaccine against SARS-CoV-2 (Ad5-S-nb2) in which the S-coding sequence of SARS-CoV-2, inserted in the E1 region of an E1/E3-deleted Ad5 vector, was optimized by altering the codon usage to efficiently express in human cells and (Feng et al., 2020). The authors claim that the great potency of Ad5-S-nb2 may enable the induction of protective immunity using low dosages what could reduce side-effects. Several other examples have shown the benefits of eliciting a high transgene-specific immune response by applying codon-usage transgene optimization (Shoji et al., 2012; Feng et al., 2018). However, in the development of DNA vaccines codon optimization did not always positively correlate with efficacy (Li and Petrovsky, 2016). The fact that mRNAs contain multiple layers of information should be carefully considered by codon optimization strategies to avoid potential undesired effects.

## Future Perspectives

The use of adenoviral vectors as vaccines against viral pathogens is continuously growing and it is at the forefront of vaccinology. While there are still some obstacles to overcome, novel approaches are being developed that expand the field.

Codon deoptimization has proven to be a useful and effective strategy for viral attenuation with different viruses. In the context of replication-competent adenovirus-based vaccines, synonymous recoding strategies have been poorly explored but codon optimization of adenoviral proteins and/or transgene antigens are showing good potential in vaccine development. Such knowledge can lead to the development of additional codon optimization strategies that can concert a good balance between transgene antigen expression and virus attenuation. However, the complexity of codon usage demands a profound understanding of adenovirus-host interactions. Moreover, vaccine manufacturing aspects should be taken into account when introducing such modifications to enable large scale productions that could meet the requirements for clinical applications. Altogether emphasizes the need for future studies that demonstrate the efficacy of transgene/adenoviral protein recoding to position this strategy as a real alternative in adenoviral-based vaccines.

## Author Contributions

JG-R, EV, EN-M, RA, and CF contributed in the manuscript writing. All authors contributed to the article and approved the submitted version.

## Conflict of Interest

The authors declare that the research was conducted in the absence of any commercial or financial relationships that could be construed as a potential conflict of interest.
